# Stabilisation of β-Catenin Downstream of T Cell Receptor Signalling

**DOI:** 10.1371/journal.pone.0012794

**Published:** 2010-09-16

**Authors:** Matthew Lovatt, Marie-José Bijlmakers

**Affiliations:** Peter Gorer Department of Immunobiology, School of Medicine at Guy's, King's College and St Thomas' Hospitals, King's College London, Guy's Hospital, London, United Kingdom; New York University, United States of America

## Abstract

**Background:**

The role of TCF/β-catenin signalling in T cell development is well established, but important roles in mature T cells have only recently come to light.

**Methodology/Principal Findings:**

Here we have investigated the signalling pathways that are involved in the regulation of β-catenin in primary human T cells. We demonstrate that β-catenin expression is upregulated rapidly after T cell receptor (TCR) stimulation and that this involves protein stabilisation rather than an increase in mRNA levels. Similar to events in Wnt signalling, the increase in β-catenin coincides with an inhibition of GSK3, the kinase that is required for β-catenin degradation. β-catenin stabilisation in T cells can also be induced by the activation of PKC with phorbol esters and is blocked by inhibitors of phosphatidylinositol 3-kinase (PI3K) and phospholipase C (PKC). Upon TCR signalling, β-catenin accumulates in the nucleus and, parallel to this, the ratio of TCF1 isoforms is shifted in favour of the longer β-catenin binding isoforms. However, phosphorylated β-catenin, which is believed to be inactive, can also be detected and the expression of Wnt target genes *Axin2* and *dickkopf* is down regulated.

**Conclusions/Significance:**

These data show that in mature human T cells, TCR signalling via PI3K and PKC can result in the stabilisation of β-catenin, allowing β-catenin to migrate to the nucleus. They further highlight important differences between β-catenin activities in TCR and Wnt signalling.

## Introduction

Wnt/β-catenin signalling is important for cell fate decisions during many developmental programs. The canonical Wnt signalling pathway is initiated upon binding of Wnt to the receptor Frizzled and its co-receptor LRP, which ultimately leads to the stabilisation and accumulation of β-catenin. Stabilised β-catenin translocates to the nucleus and associates with the transcription factors TCF and LEF to drive transcription of Wnt regulated genes [1], [2]. In the absence of a Wnt signal, β-catenin associates with a destruction complex comprising the kinases glycogen synthase kinase 3 (GSK3) and casein kinase 1 (CK1), and the scaffolding proteins Axin and adematosis polyposis coli (APC). This interaction results in the phosphorylation of β-catenin at its N-terminus by GSK3/CK1, which serves as a recognition signal for ubiquitination by the SCF E3 ligase βTrCP and leads to the degradation of β-catenin by the proteasome [2]. Thus, the regulation of β-catenin stability is key to Wnt signalling. Mutations in the N-terminal phosphorylation sites of β-catenin and in the β-catenin destruction complex proteins Axin and APC are found in multiple cancers, suggesting that strict regulation is essential to avoid malignancies [2].

Wnt/β-catenin signalling regulates many aspects of T cell development [3], [4] but its role in mature T cells is less clear. Early reports suggested a lack of β-catenin expression and transcriptional activity in peripheral human T cells [5] and a failure of GSK3 inhibition to induce TCF/β-catenin dependent transcription in the Jurkat T cell line [6], [7]. However, recent data have demonstrated several important roles for TCF1/β-catenin in mature T cell differentiation and function. For murine CD4+ T cells, the expression of high levels of a stable form of β-catenin in T_reg_ cells was shown to increase cell survival, resulting in an enhanced protection against inflammatory bowel disease in a mouse model [8]. In the same report it was demonstrated that retroviral expression of stable β-catenin in naïve CD4^+^ T cells renders these cells anergic [8]. More recently, Sen and co-workers [9] have shown that TCF1 and β-catenin play a critical role in T_H_2 differentiation. TCF1/β-catenin were found to activate the transcription of GATA-3-1b early after TCR activation. Furthermore, in activated effector T cells, β-catenin has been shown to regulate expression of matrix metalloproteinases MMP2 and MMP9 during T cell extravasation, which promotes migration through subendothelial basement membrane [10]. Finally, several studies have demonstrated an important role for TCF1/β-catenin in the generation of functional CD8+ memory T cells in mice [11], [12], [13]. Most notably, the expression of a stabilised β-catenin transgene was shown to promote the induction of CD8+ memory T cells, whereas the absence of TCF1 or β-catenin resulted in a defect in central CD8+ memory T cell differentiation [13]. Consistent with a role for TCF1/β-catenin in mature T cells, a dynamic regulation of the multiple isoforms of TCF that arise from alternative splicing and alternative promoter usage [14] upon activation of naïve and memory CD8^+^ T cells has also been demonstrated [15]. Despite these reports there is little information on how β-catenin is regulated in T cells, but studies on immature and mature mouse T cells have suggested that pre-TCR and TCR signalling can stabilise β-catenin [16], [17], [18], [19]. An obvious player in this pathway is GSK3, an unusual kinase that it is constitutively active in the absence of a signal. TCR signalling is known to inhibit GSK3 and this controls the localisation of the transcription factor NFAT in the nucleus [20]. Here, we have examined the expression pattern of β-catenin in primary human T cells following activation through the TCR. We report that β-catenin is post-transcriptionally regulated by TCR signalling and that this occurs in a protein kinase C (PKC) and phosphatidylinositol 3-kinase (PI3K) dependent fashion.

## Results

### TCR signalling up-regulates β-catenin in primary human T Cells

**Figure 1:**
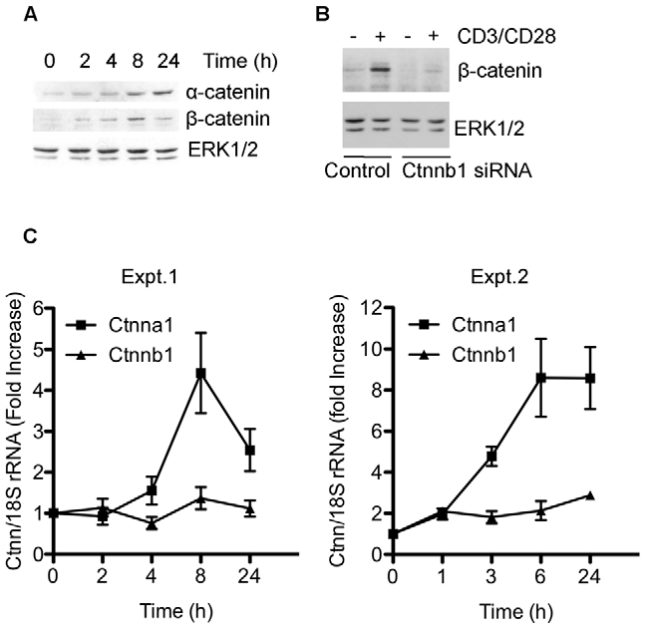
TCR signalling increases the level of β-catenin. T cells were stimulated with anti-CD3 antibodies for the indicated times. (A) Cell aliquots were analysed for expression of α-catenin or β-catenin protein levels by Western blotting. Western blotting with anti-ERK was used to control for protein loading. (B) Antibody specificity was confirmed by transfecting T cells with control RNAi (Luciferase) or RNAi directed against β-catenin (Ctnnb1) prior to T cell stimulation for 18 hours. (C) mRNA levels for α-catenin (Ctnna1) and β-catenin (Ctnnb1) were measured by qPCR relative to expression of 18srRNA (endogenous control). Two independent experiments are shown for comparison. Experiments were performed in triplicate with similar results obtained when compared against alternative endogenous controls actin, GAPDH and HPRT (not shown). The cells used in Expt1 were used for Western blotting in (A).

Signalling via the TCR in combination with CD28 is known to inhibit GSK3 [21] which could lead to an increase in the expression of β-catenin. To investigate this, human primary T cells were isolated, stimulated with agonist antibodies to the TCR complex and β-catenin expression was analysed by Western blotting. β-catenin was virtually undetectable in unstimulated cells, but the protein was upregulated as early as 2 hours after TCR stimulation (Figure 1A). The levels of β-catenin further increased with time, peaked around 8 hours, and were still enhanced after 72 hours of stimulation (Figure 1A). This increase in β-catenin was efficiently abolished when T cells were transfected with siRNA against β-catenin prior to T cell stimulation (Figure 1B). Thus, these experiments reveal that β-catenin is rapidly up-regulated in response to TCR stimulation.

To investigate whether β-catenin up-regulation occurs at the mRNA or protein level, we monitored β-catenin mRNA levels in stimulated T cells by qRT-PCR. T cell activation had little impact on β-catenin mRNA levels over a range of time points (Figure 1C), despite increased protein expression within the same population of T cells (Figure 1A). Therefore, we conclude that up-regulation of β-catenin downstream of TCR signalling does not result from an increase in β-catenin mRNA levels, but reflects changes in post-transcriptional events such as protein translation or protein stability. During the course of these experiments, we also considered the expression of α-catenin and found that levels of this protein also increased over time following TCR stimulation (Figures 1A). However, in contrast to the result with β-catenin, the enhanced α-catenin protein levels resulted from an increase in mRNA levels as detected by qRT-PCR (Figure 1C).

### β-catenin protein levels are enhanced through GSK3 inactivation

**Figure 2:**
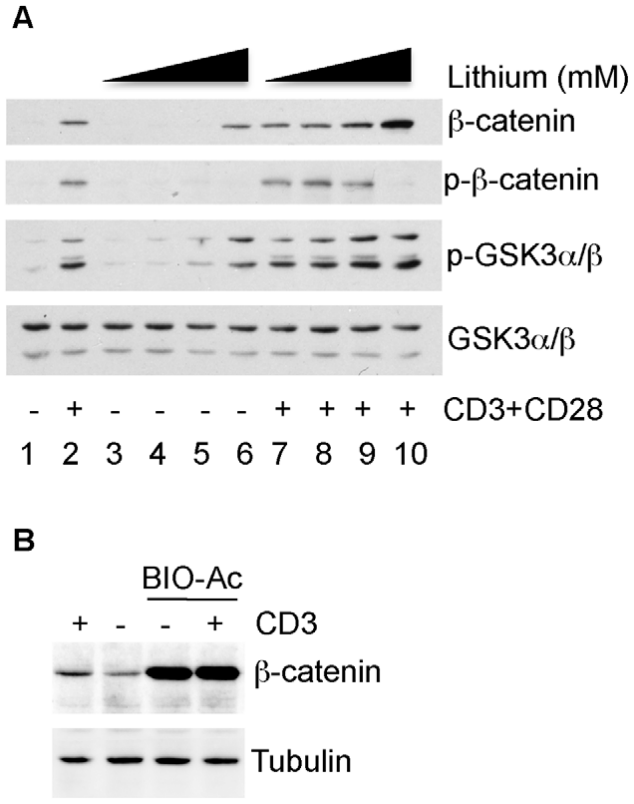
GSK3 regulates the expression of β-catenin downstream of TCR signalling. (A) Primary human T cells were left unstimulated (lane 1) or stimulated for 18 hours with plate bound anti-CD3 and anti-CD28 antibodies (lane 2), or with increasing concentrations of LiCl (0, 0.2, 2 or 20 mM, indicated by black triangles, lanes 3–6), or with anti-CD3/CD28 and increasing LiCl concentrations together (lanes 7–10). Total cell lysates were immunoblotted with antibodies specific for β-catenin, phospho-β-catenin (Ser33/37/Thr41, herein referred to as p-β-catenin), phospho-GSK3α/β (Ser21/9, herein referred to as p-GSK3α/β) and GSK3. (B) T cells were stimulated for 18 hours with plate-bound anti-CD3 as indicated, either in the absence or presence of the GSK3 inhibitor Bio-ac at 1 µM. Total cell lysates were analysed by Western blot with anti-β-catenin. An anti-tubulin blot is shown as a control for protein loading. This experiment was repeated twice with similar results.

**Figure 3:**
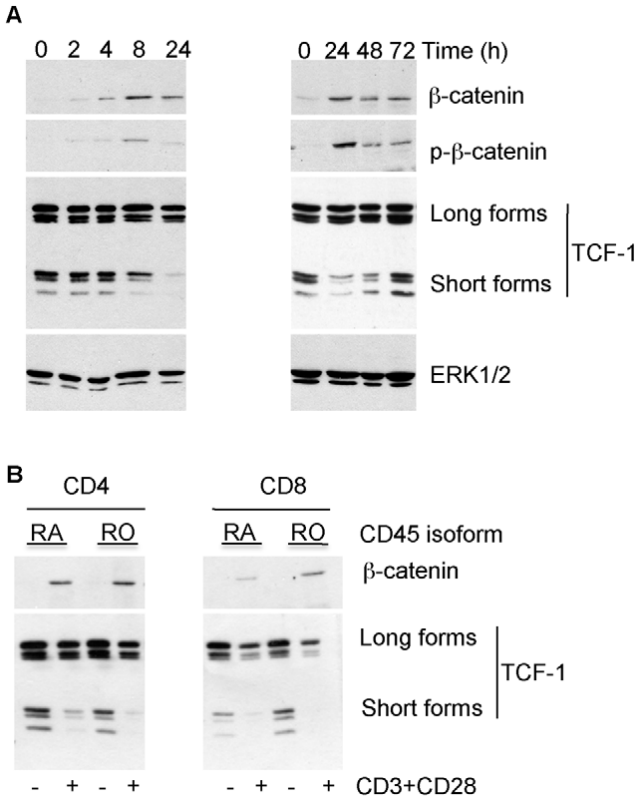
Induction of β-catenin correlates with a modulation of TCF1 isoform usage. (A) T cells were stimulated with plate bound anti-CD3 and anti-CD28 antibodies for the indicated times and immediately frozen as dry pellets on dry ice. Total cell lysates were prepared and run on SDS-PAGE, immunblotted for β-catenin, phospho-β-catenin and TCF-1. Total ERK expression served as a loading control. (B) T cells were fractionated based on the expression of CD4 and CD8 as well as CD45 isoform expression (CD45RA: naïve T cells or CD45RO: memory T cells) as outlined in materials and methods. The cells were either left unstimulated or were stimulated overnight with anti-CD3/CD28 antibodies. Total cell lysates were immunoblotted for β-catenin and TCF1.

In the absence of a signal, levels of β-catenin are low because of its phosphorylation on residues Ser33/37 and Thr41 [2] by the constitutively active kinase GSK3, which targets β-catenin for ubiquitination and subsequent proteasomal degradation. Wnt signalling leads to the inactivation of GSK3 and thus the accumulation of active, nuclear β-catenin. We investigated whether similar events are involved in the increase in β-catenin levels in response to anti-CD3/CD28 antibody stimulation. GSK3 inhibition was monitored by GSK3 phosphorylation on inhibitory residues Ser21 (GSK3α) and Ser9 (GSK3β) as this is known to correlate with a reduction in GSK3 activity [22]. Stimulation with anti-CD3/CD28 antibodies indeed resulted in increased phosphorylation of GSK3α/β (Figure 2, lane 2). As a control in these experiments we included lithium, which directly inhibits GSK3 [23]. The levels of β-catenin protein detected following overnight stimulation with anti-CD3/CD28 were comparable to those in cells treated with 20 mM lithium (Figure 2, compare lanes 2 and 6), a standard concentration reported to stabilise β-catenin in other cell types [24]. Consistent with this, the phosphorylation of GSK3α/β in CD3/CD28 stimulated cells was also comparable to that in T cell cultures exposed to 20 mM lithium (Figure 2, lanes 2 and 6). Unexpectedly, despite the apparent inhibition of GSK3α/β after T cell stimulation, β-catenin phosphorylated on Ser33/37 and Thr41 was detectable in anti-CD3/CD28 stimulated cells (Figure 2, lane 2). Moreover, time course experiments showed that these phosphorylated forms appear with similar kinetics as total β-catenin (see Figure 3A).

The presence of lithium and anti-CD3/28 stimulation together enhanced β-catenin levels relative to that observed with either anti-CD3/CD28 or lithium alone (Figure 2, lane 10 versus lanes 2 and 6). The combined presence of anti-CD3/CD28 and lithium also further increased the phosphorylation of GSK3 and led to an absence of phosphorylated β-catenin (Figure 2, lane 10). This additive effect of lithium suggests that GSK3 may not be completely inhibited following stimulation via the TCR. However, lithium has a dual effect on GSK3: it directly inhibits GSK3 activity by competing with Mg^2+^ and can further enhance GSK3 inhibition by reducing the activity of phosphatases that dephosphorylate Ser21 (GSK3α) and Ser9 (GSK3β) [23], [25]. Moreover, lithium has been shown to inhibit other protein kinases as well. Therefore, we also compared the effect of TCR activation on β-catenin levels with that of an alternative inhibitor of GSK3, the ATP-competitor BIO-acetoxime (BIO-ac, also called GSK3 inhibitor X). BIO-ac is a more selective analogue of 6-bromoindirubin-32-oxime (BIO) that exhibits greater selectivity for GSK3 than lithium. BIO-ac robustly induced β-catenin stabilisation in primary human T cells. However, T cell activation in combination with BIO-ac had no additive effect on β-catenin levels. This likely reflects the greater potency of BIO-ac over lithium, resulting in maximum inhibition of GSK3 and therefore greater induction of β-catenin levels.

In summary, we conclude that the increase in β-catenin levels upon TCR signalling correlates with GSK3 inhibition, suggesting that it results from a stabilisation of the β-catenin protein. The residual phosphorylation of this stabilised β-catenin may arise from an incomplete inactivation of GSK3.

### The induction of β-catenin occurs in parallel with a modulation of TCF isoforms

Primary T cells have been shown to express full length and short isoforms of the transcription factor TCF1 in equivalent amounts (Figure 3A) [15]. Of these, the N-terminally truncated, short TCF1 isoforms can bind to DNA but cannot interact with β-catenin and are known to antagonise β-catenin signalling [14]. When primary human T cells were activated through the TCR, a gradual decrease in the relative levels of the short TCF1 isoforms was observed with time, resulting in the virtual absence of these forms at 24 hours post stimulation (Figure 3A). This down-modulation was transient and a complete re-expression of short TCF1 isoforms was seen at 72 hours after TCR activation.

To explore the stabilisation of β-catenin and modulation of TCF1 isoforms further, we next isolated populations of CD4+ and CD8+ T cells and divided these into naïve and memory subsets based on the surface expression of CD45 isoforms (Figure 3B). Stimulation via the TCR showed that levels of β-catenin were similarly induced in CD4+ and CD8+ T cells and that this increase in β-catenin was also irrespective of whether naïve (CD45RA+) or memory (CD45RO+) T cells were studied. Moreover, the expression and modulation of TCF1 isoforms following activation was also observed for all T cell population to a similar extent (Figure 3B). Taken together we conclude that T cell activation stabilises β-catenin and changes the ratio of TCF1 isoforms in favour of the full length, β-catenin binding isoforms.

### TCR signalling drives nuclear accumulation of β-catenin but target genes are downmodulated

**Figure 4:**
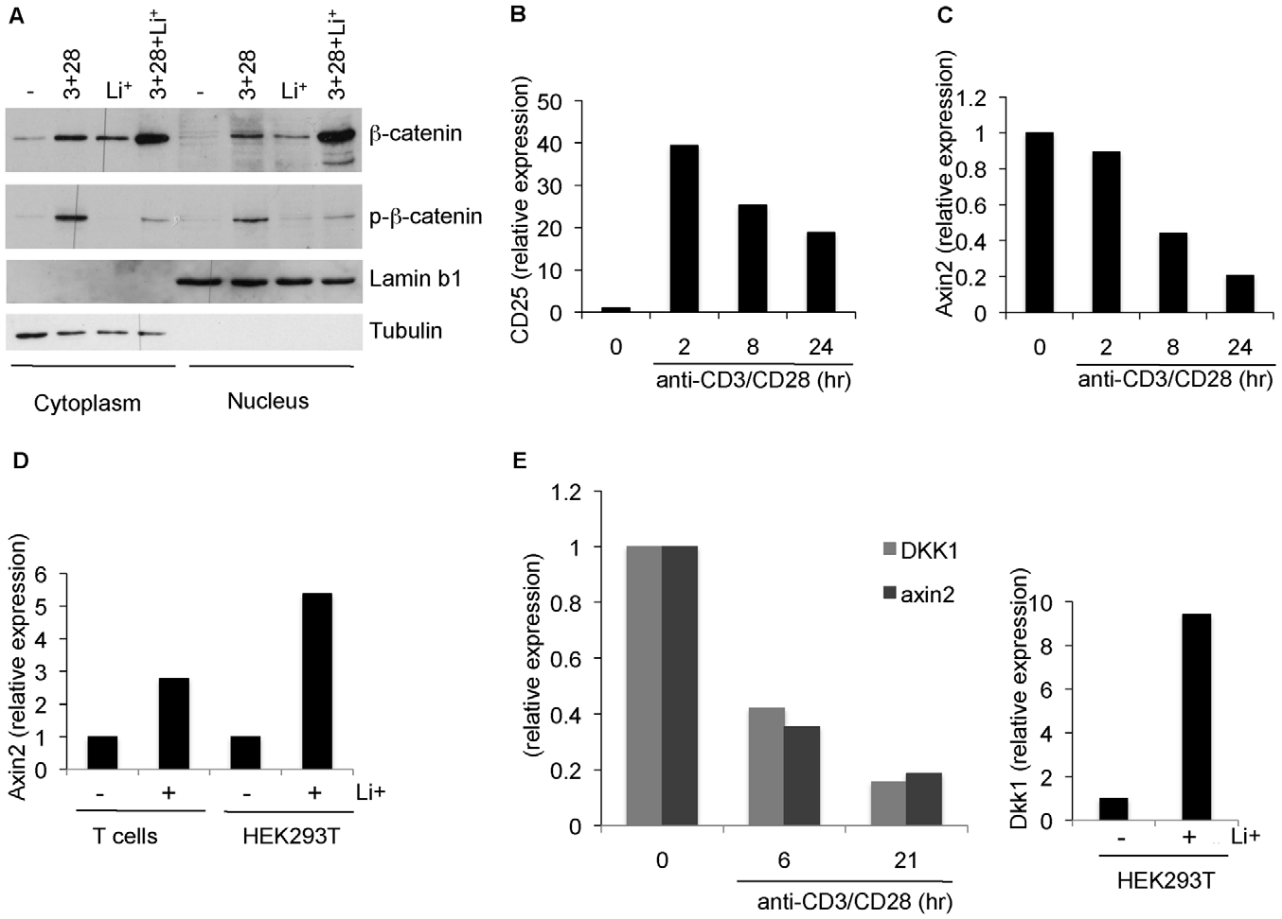
TCR stimulation leads to nuclear translocation of β-catenin. (A) T cells were stimulated with plate bound anti-CD3/CD28 in the presence or absence of 20 mM LiCl for 18 hours, as indicated, following which cytoplasmic and nuclear fractions were separated. Fractions were immunoblotted for β-catenin and phospho-β-catenin expression. Cytoplasmic and nuclear fractionation was confirmed by tubulin (cytosol) and lamin-B1 (nuclear) expression. (B) T cells were stimulated with anti-CD3/CD28 magnetic beads for the indicated times. CD25 (IL-2Rα) mRNA levels were measured by qRT-PCR, using Abl1 as an internal control, to verify successful T cell activation. (C) The expression of the TCF/β-catenin target gene *Axin2* was measured by qRT-PCR following stimulation of T cells with anti-CD3/CD28 (same samples as in B). (D) In parallel to the samples in B and C, T cells and HEK293T cells were left untreated or incubated with 20 mM lithium for 7 hours and measured for Axin2 mRNA levels relative to those of Abl1. (E) Left: T cells were stimulated with anti-CD3/CD28 for the indicated times and Axin2 and DKK-1 mRNA levels were determined by qRT-PCR relative to Abl and PPIA. The expression relative to Abl1 is shown, with calculations relative to PPIA yielding comparable results. Right: HEK293T cells were left untreated or incubated with 20 mM lithium for 7 hours and measured for DKK-1 mRNA levels relative to those of Abl1. All qRT-PCR reactions were performed in triplicate.

When β-catenin is stabilised following Wnt signalling, the de-phosphorylated ‘active’ β-catenin translocates to the nucleus and associates with TCF/LEF proteins to drive transcription of TCF/LEF dependent genes. We explored whether nuclear translocation of β-catenin also occurred following T cell activation. Following overnight incubation with various stimuli, T cell homogenates were separated into cytoplasmic and nuclear fractions, the purity of which was demonstrated by Western blotting for the cytoplasmic protein tubulin and the nuclear protein lamin B1 (Figure 4). In T cells stimulated with anti-CD3/CD28, a considerable fraction of β-catenin could be detected in the nucleus, similar to that observed following lithium treatment (Figure 4). The presence of both anti-CD3/28 and lithium resulted in the highest relative amount of β-catenin in the nucleus. When cells were stimulated with anti-CD3/CD28, phosphorylated forms of β-catenin were found in the nucleus, which is surprising given that β-catenin is thought to be active in the dephosphorylated form [26]. Nevertheless, these results show that like Wnt signalling, TCR signalling leads to nuclear localisation of β-catenin. To monitor whether this presence of β-catenin in the nucleus resulted in an increase in TCF/β-catenin transcriptional activity, we measured induction of known Wnt target genes at several time points after T cell activation (list of Wnt target genes available from http://www.stanford.edu/~rnusse/wntwindow.html). A robust increase in mRNA levels of CD25 (the IL-2 receptor α-chain) with anti-CD3/CD28 stimulation, demonstrated that efficient T stimulation had indeed occurred (Figure 4B). We initially studied the expression of *Axin2* as a well-characterized TCF/β-catenin target. Surprisingly, T cell activation resulted in the down modulation of Axin2 mRNA levels as measured by qRT-PCR (Figure 4C). On the other hand, the parallel treatment of T cells with lithium resulted in an up-regulation of Axin2 mRNA levels, comparable to that in HEK293T cells, indicating that a TCF/β-catenin dependent increase in expression of this gene does occur in primary human T cells (Figure 4D). Similarly, we found that in a separate experiment another target of Wnt/TCF signalling, Dickkopf (DKK-1) was also down regulated by TCR signalling (Figure 4E, left), despite this gene being upregulated in lithium-treated HEK293T cells (Figure 4E, right). We conclude that T cell activation appears to affect certain TCF/β-catenin target genes resulting in their down regulation. It is noted that both Axin2 and DKK-1 are known negative regulators of Wnt signals.

### PKC and PI3K regulate GSK phosphorylation and β-catenin levels downstream of the TCR

**Figure 5:**
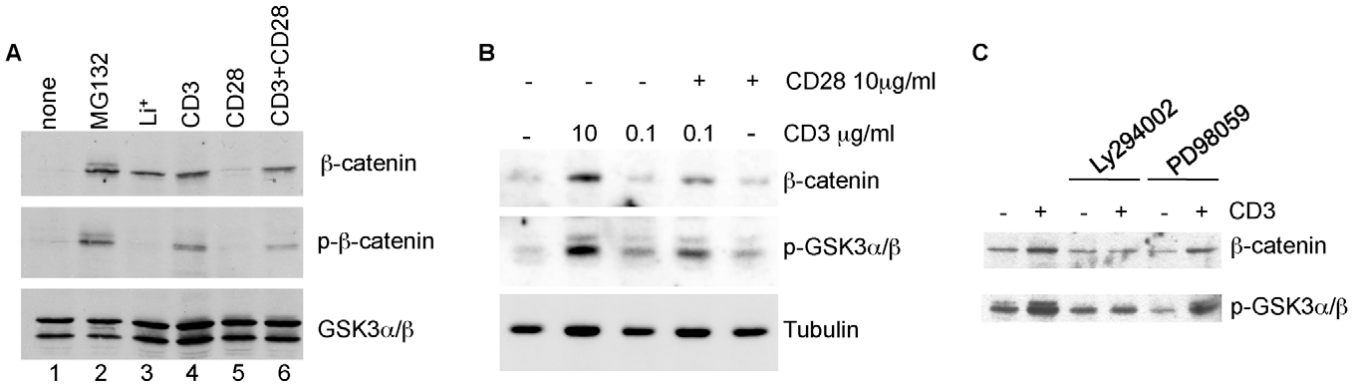
PI3K regulates the expression of β-catenin downstream of TCR signalling. (A) T cells were left untreated or were incubated for 18 hours with the proteasome inhibitor MG132, 20 mM LiCl, plate bound anti-CD3 (10 µg/ml), plate bound anti-CD28 (10 µg/ml), plate bound anti-CD3 plus anti-CD28 (10 µg/ml each). Total cell lysates were used for Western blotting with anti-β-catenin, anti-phospho-β-catenin and anti-GSK3αβ. (B) T cells were stimulated for 18 hours with two different concentrations of plate-bound anti-CD3 in the absence or presence of anti-CD28. Total cell lysates were analysed by Western blot with anti-β-catenin, anti-p-GSK3αβ and anti-tubulin. This experiment was performed twice with similar results. (C) T cells were pre-treated with the PI3K inhibitor Ly294002 (50 µM) or the MEK inhibitor PD98059 (50 µM) for 1 hour prior to 18 hours stimulation with plate bound anti-CD3 in the presence of these inhibitors. Total cell lysates were immunoblotted with indicated antibodies.

To probe further the signals required to induce β-catenin up-regulation in T cells, T cell cultures were treated with a variety of compounds and stimuli (Figure 5A). The addition of the proteasome inhibitor MG132 increased levels of β-catenin as well as that of phospho-β-catenin (Figure 5A, lane 2), consistent with the notion that β-catenin levels are normally low because of proteasomal degradation upon phosphorylation by GSK3. Next, the effect of stimulation with either anti-CD3 or anti-CD28 separately was compared to that of anti-CD3 and anti-CD28 combined. The results showed that anti-CD3 stimulation at 10 µg/ml is sufficient to increase β-catenin levels (Figure 5A, lane 4). Anti-CD28 on its own had no effect (Figure 5A, lane 5) nor did it increase the amount of β-catenin induced in the presence of this concentration of anti-CD3 (Figure 5A, compare lanes 4 and 6). Levels of β-catenin phosphorylation were also similar for anti-CD3 and anti-CD3/CD28 stimulated T cells (Figure 5A, lanes 4 and 6). However, the use of this high concentration of plate bound anti-CD3 antibody could have masked potential effects of CD28 activation. Therefore, we repeated these experiments using graded doses of plate bound anti-CD3 stimulation. Interestingly, effects of anti-CD28 could be visualised when the concentration of CD3 was reduced to 1 µg/ml (not shown) or 0.1 µg/ml (Figure 5B), resulting in higher β-catenin levels in the presence of anti-CD28.

**Figure 6:**
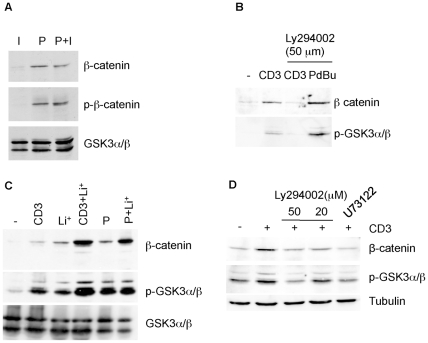
PKC can also regulate the expression of β-catenin. (A) T cells were incubated for 18 hours with either Ionomycin (I, 100 nM), PDBu (P, 10 ng/ml), or a combination of PDBu and Ionomycin (P+I). Total cell lysates were used for Western blotting with anti-β-catenin, anti-phospho-β-catenin and anti-GSK3. (B) T cells were stimulated for 18 hours with either anti-CD3 or PDBu in the absence or presence of the PI3K inhibitor Ly294002. Total cell lysates were analysed by Western blot with anti-β-catenin and anti-phospho-GSK3. (C) T cells were stimulated for 18 hours with either plate-bound CD3 (CD3), lithium (Li+) or PDBu (P), as shown, and analysed by Western blot with the antibodies indicated. (D) T cells were stimulated with anti-CD3 for 18 hours either in the absence or presence of the PI3K inhibitor Ly294002 (20 and 50 µM) or the PLC inhibitor U73122 (10 µM). Total cell lysates were analysed by Western blot with anti-β-catenin, anti-p-GSK3α/β and anti-tubulin. A representative figure of two experiments is shown.

CD28 ligation initiates PI3K activation [27], which activates AKT leading to GSK3 phosphorylation and inactivation. Consistent with this, higher levels of pGSK3 phosphorylation were observed in the presence of anti-CD28 when cells were stimulated with a low dose of anti-CD3 (Fig. 5B). Robust stimulation with anti-CD3 alone is also known to induce PI3K dependent AKT activation and consequently GSK3 inactivation [28]. To determine whether activation of PI3K plays a role in the increase in β-catenin levels after T cell activation, T cells were treated with the PI3K inhibitor LY294002 before and during stimulation. Indeed, LY294002 treatment reduced anti-CD3 induced GSK3 phosphorylation downstream of PI3K (Figure 5C, see also Figure 6B, D) as well as AKT phosphorylation (not shown). Moreover, the increase in β-catenin levels was also drastically reduced in the presence of this inhibitor (Figure 5C, Figure 6 B, D). On the other hand, the MEK inhibitor PD98059 had no effect on the induction of either GSK3 phosphorylation or β-catenin levels (Figure 5C). These data suggest that TCR signalling via PI3K/AKT and the resulting inactivation of GSK3 lead to the stabilisation of β-catenin.

An induction of β-catenin levels could also be observed by mimicking signalling downstream of the TCR with a combination of the phorbol ester PDBu, a potent activator of PKC isoforms, and the calcium ionophore ionomycin (Figure 6A). Separate stimulation with either PDBu or ionomycin showed that treatment with PDBu alone was sufficient to enhance β-catenin levels (Figure 6A). Importantly, this PDBu mediated stabilisation of β-catenin was not affected by the presence of the PI3K inhibitor LY294002 (Figure 6B). PDBu treatment is known to induce GSK3 phosphorylation in a variety of cell types including B cells [29] and similarly, treatment of T cells with PDBu induced GSK3 phosphorylation (Figure 6C). However, despite this apparent GSK3 inhibition, phosphorylated β-catenin proteins were detectable following stimulation with PDBu (Figure 6A), as was previously observed for cells stimulated with anti-CD3/CD28 (Figures 2A, 3A and 4A). Also similar to anti-CD3/CD28 stimulation, the effect of PDBu on β-catenin could be increased by the addition lithium (Figure 6C). These effects of PDBu suggest that PKC may also play a role in the stabilisation of β-catenin downstream of TCR signalling. PDBu mimics diacylglycerol (DAG), which is generated from the actions of phospholipase C (PLC) on phosphatidylinositol 4,5-bisphosphate (PIP2), in the activation of DAG-dependent isoforms of PKC. To address the role of PKC in β-catenin stabilisation, and to avoid the use of PKC inhibitors that may also target GSK3, we treated T cell cultures with the PLC inhibitor U73122. T cell activation in the presence of U73122 resulted in the inhibition of β-catenin stabilisation comparable to that achieved by PI3K inhibition with 50 µM Ly294002 (Figure 6D). Thus, this result indicates that downstream of the TCR, PLC-DAG activates PKC resulting in an increase in β-catenin levels. Taken together these data suggest that TCR signalling via both PI3K and PKC play a role in the inactivation of GSK3, which in-turn leads to the stabilisation of β-catenin.

## Discussion

We explored the regulation of β-catenin expression in primary human T cells and show that like in other cell types, β-catenin levels are low in the absence of a signal owing to proteasome-mediated degradation. However, upon T cell stimulation, a rapid, post-transcriptional upregulation of β-catenin takes place, similar to events that occur upon Wnt signalling. Activation of both PI3K and PKC can induce the increase in levels of β-catenin, which coincides with the phosphorylation and therefore inhibition of GSK3, the kinase that normally targets β-catenin for degradation. We further show that stabilised β-catenin can be detected in the nucleus. Moreover, the expression of TCF1 isoforms were found to be differentially regulated so that the longer β-catenin-interacting forms predominate in the nucleus following T cell activation. These data are consistent with important activities of TCF/β-catenin in mature T cells such as the recently described functions in T cell migration T_reg_ function [8], memory T cell differentiation [11], [12], [13], and the regulation of GATA3 expression during T_H_2 differentiation [9].

Although effects of Wnt on β-catenin in pre-activated T cells have been demonstrated [10], our data indicate that β-catenin can be regulated directly downstream of TCR signalling. Firstly, we show that β-catenin up-regulation is rapid and can be detected within 2 hrs of T cell activation (Figures 1 and 3). The involvement of Wnts in this scenario would require an induction of *wnt* genes downstream of the TCR and the subsequent signalling through Frizzled receptors, which is unlikely to occur within this time frame. Secondly, we show that β-catenin upregulation can be blocked by Ly29004 (Figure 5), an inhibitor of PI3K that is activated downstream of the TCR and is not thought to be involved in Wnt signalling [30]. Furthermore, we did not observe effects on β-catenin levels when biologically active Wnt3a conditioned media or recombinant Wnt3a was added to freshly isolated unstimulated cells (data not shown). Nevertheless, it is possible that external Wnt can cooperate with TCR signals at later stages and augment the inhibition of GSK3. Similar to the additive effects observed with lithium (Figure 2) [24], this could further enhance β-catenin levels and decrease β-catenin phosphorylation. Indeed, Wnt3a has been reported to stabilise β-catenin in effector T cells and inhibit the proliferation of activated CD8+ T cells [11].

PI3K appears to play a role in the stabilisation of β-catenin via the activation of AKT that in turn inactivates GSK3 proteins. The fact that Jurkat T cells, which are deficient in the PIP3 phosphatase PTEN that inactivates AKT, express high levels of β-catenin compared to primary T cells is entirely consistent with this finding [5, and data not shown]. Furthermore, the dysregulation of AKT may also explain the lack of lithium induced TCF/β-catenin transcriptional activity that has been reported for Jurkat cells [7]. We demonstrate that the activation of PKC by phorbol esters can increase β-catenin levels as well, and that PLC inhibitors inhibit this induction (Figure 6). Interestingly, such a dual involvement of PI3K and PLC/PKC was also demonstrated for the stabilisation of β-catenin in B cells following B cell receptor triggering [29] and may reflect a role of PI3K in the activation of PLC. For both PI3K and PKC pathways, the inactivation of GSK3 appears to be the central event in the stabilisation of β-catenin.

A surprising observation was the presence of phosphorylated β-catenin following T cell activation. The fact that the GSK3 inhibitor lithium blocked this phosphorylation suggests that TCR signalling does not completely inactivate GSK3. Indeed, it has been shown that the combination of phorbol ester and ionomycin decreases GSK3 activity by approximately 50% in T cells, and that this inhibition is transient despite persistent stimulation [7], [31]. Unexpectedly, the phosphorylated forms of β-catenin were detected over a long period of time (72 hours, Figure 3) even though N-terminal phosphorylation normally targets β-catenin for ubiquitin-mediated proteasomal degradation. It is possible that T cell activation inhibits to some extent components of the β-catenin destruction pathway, but this has not yet been established. Another unexpected finding was the presence of phosphorylated β-catenin in nuclear fractions (Figure 4). This could result from the nuclear translocation of phosphorylated β-catenin, or the phosphorylation of β-catenin in the nucleus by GSK3, which has been demonstrated to regulate NFAT [20] and CyclinD1 [32] in this compartment. The role of this nuclear phosphorylated β-catenin is not yet clear. It has been shown that phosphorylated β-catenin can interact with LEF-1 but cannot form a ternary complex with DNA [33] and cannot drive TCF reporter activity [26]. However, a functional role of phosphorylated β-catenin in the nucleus remains possible as well. Indeed, one study has demonstrated that phosphorylated β-catenin localises to centrosomes and may regulate the microtubule organizing centre [34]. Despite several attempts using Western blotting and immunofluoresence we were unable to detect active β-catenin with an antibody specific to the non-phosphorylated protein (data not shown). We do not exclude that active non-phosphorylated β-catenin is present but the levels of this protein may be below the detection limits of this reagent. Nevertheless, the presence of phosphorylated nuclear β-catenin indicates that different pathways are operational downstream of TCR signalling compared to Wnt signalling.

Transcriptional activity of TCF1/β-catenin early after T cell stimulation has been demonstrated [9]. Consistent with this, we found that TCF1 transcripts are regulated upon T cell activation so that the β-catenin binding forms become the most abundant (Figure 3 and [14]). Surprisingly, our data also revealed that mRNA levels of the TCF/β-catenin target genes *Axin2* and *DKK-1* were reduced upon TCR activation. Axin2 and DKK-1 are inhibitors of Wnt signalling and it is possible that an active inhibition of these antagonists renders T cells more sensitive to Wnt signalling at a later stage after T cell activation. Whereas this remains to be determined, the down regulation of these TCF/β-catenin targets again clearly demonstrates that the functions of β-catenin after TCR signalling do not necessarily mimic those described for the Wnt signalling pathway.

Another β-catenin partner, α-catenin, was also found to be upregulated upon TCR signalling (Figure 1), which to our knowledge has not been described before. A complex of α-catenin and β-catenin plays a role in cell-cell adhesion downstream of the adhesion molecule cadherin. In the absence of an established role for cadherin in T cells, it will be interesting to determine the function of this enhanced α-catenin. Interestingly, α-catenin knockout keratinocytes display hyperpoliferative responses with hyperactive RAS/MAPK signalling, suggesting that α-catenin may play a role in processes other than adhesion [35].

In summary, our data provide new insights into the regulation of β-catenin in response to TCR signalling in peripheral human T cells, and reveal remarkable discrepancies between the activity of TCF/β-catenin in Wnt and TCR signalling. This provides an important basis for further studies into the various roles that have emerged for β-catenin in mature T cells, keeping in mind that these functions may also to a large extent be influenced by the differentiation state of the cells.

## Materials and Methods

### Reagents

Chemicals were from Sigma-Aldrich unless otherwise indicated.

### T cell isolation, stimulation and drug treatment

Primary human PBMCs were isolated from buffy coats (National Blood Service) using Lymphoprep (Axis-Shield) and density-gradient centrifugation. T cells were purified on nylon wool (Kisker) columns yielding about 85–90% pure populations. T cells were maintained in RPMI-1640 (Invitrogen), 10% FCS (Helena Biosciences) and the antibiotics Penicillin (100 U/ml)/Streptomycin (0.1 mg/ml streptomycin) (Gibco Ltd.) and used for experiments within 24 hours of isolation. Alternatively, nylon wool purified T cells were immunostained with APC conjugated anti-CD4, PerCP conjugated anti-CD8, FITC conjugated anti-CD45RA and PE conjugated anti-CD45RO (eBiosciences) on ice for 1 hour in PBS 0.2% BSA. Cells were sorted on Becton Dickinson Aria Flow Cytometer and immediately processed.

T cells (2–5×10^6^) were stimulated with plate-bound anti-CD3 and anti-CD28 antibodies (10 µg/ml each, unless otherwise stated) for the indicated times. Where applicable, inhibitors of PI3K (LY29002, Cell Signalling Technology), PLC (U73122, Calbiochem), GSK3 (Bio-acetoxime, also called GSK-3 inhibitor X, Calbiochem) and MEK1/2 (PD98059, Calbiochem) were added 1 hour prior to stimulation and remained present during stimulation. PDBu was used at 10 ng/ml and ionomycin at 100 nM. LiCl was added from a 8M stock solution to the indicated final concentrations.

### RNAi

To confirm antibody specificity, T cells were transfected at 5×10^6^ cells with 50 pmoles ON-TARGET plus siRNA targeting human β-catenin (Ctnnb1), or control (Luciferase) siRNA reagents (Thermo Fisher Scientific, Lafayette, CO). T cell transfections were performed using the human T cell nucleofector reagent (Amaxa biosystems) and programme U14 of the Amaxa nucleofector. Cells were stimulated and processed 18 hours after transfections.

### SDS-PAGE and Western blotting

Antibody stimulated T cells were removed from plates with PBS containing 5 mM EDTA and lysed in ice cold lysis buffer (1% NP-40, 50 mM Tris-Cl (pH 7.5), 150 mM NaCl, 10 mM NaF, 10 mM disodium pyrophosphate) together with a cocktail of small protease and phosphatase inhibitors (Roche). Equal amounts of post-nuclear supernatants (BCA assay) were subjected to SDS-PAGE and Western blotted with indicated antibodies and visualised with enhanced chemiluminescence (Pierce). To separate nuclear and cytoplasmic fractions, T cells were lysed using the NE-PER Extraction Reagents (Pierce) according to the manufacturer's instructions.

### Antibodies

The following antibodies were used for immunoblotting: anti-β-catenin, anti-phospho-β-catenin (Ser33/37/Thr41), anti-TCF-1, anti-phospho-GSK3αβ (Ser21/9), anti-phospho-AKT (Ser473) (all Cell Signaling Technology), anti-GSK3αβ (Biosource), anti-lamin B1, anti-ERK1 (Santa Cruz Biotechnology), anti-tubulin (Invitrogen) and rabbit anti-α-catenin (VB1, [36] kind gift of V. Braga, Imperial College, London, UK).

### Quantitative real time PCR (qRT-PCR)

Isolated T cells were stimulated with plate bound anti-CD3 or anti-CD3/CD28 magnetic beads (Dynal) for the indicated time points, washed in cold PBS, pelleted and snap frozen in liquid nitrogen as dry cell pellets. Total RNA was isolated using Trizol (Invitrogen) and equivalent amounts reverse transcribed with the high capacity cDNA reverse transcription kit (Applied Biosystems). PCR was performed in triplicate in TaqMan Universal PCR master mix containing gene specific probes or endogenous control (Applied Biosystems). Ctnna1 (α-catenin) and Ctnnb1 (β-catenin) gene expression were analysed using the ABI PRISM 7900HT instrument and samples normalised to expression of endogenous control. Fold increase was calculated by the ΔΔCt method. Axin2 and DKK-1 mRNA levels were analysed using SYBR green PCR mastermix (Applied Biosystems) and primers (Eurofins MWG Operon) designed using the ProbeFinder software (Roche Applied Science). Abl1 and PPIA were used as reference genes.
